# Maternal Methyl-Donor Micronutrient Supplementation During Pregnancy Promotes Skeletal Muscle Differentiation and Maturity in Newborn and Weaning Pigs

**DOI:** 10.3389/fnut.2020.609022

**Published:** 2020-11-30

**Authors:** Qin He, Tiande Zou, Jun Chen, Li Jian, Jia He, Yingying Xia, Fei Xie, Zirui Wang, Jinming You

**Affiliations:** ^1^Key Laboratory of Animal Nutrition in Jiangxi Province, Jiangxi Agricultural University, Nanchang, China; ^2^Key Innovation Center for Industry-Education Integration of High-Quality and Safety Livestock Production in Jiangxi Province, Nanchang, China

**Keywords:** pregnancy, colostrum, offspring, myogenesis, muscle differentiation

## Abstract

Adequate maternal methyl-donor micronutrient (MET) intake is an important determinant of the organ development and metabolic renovation of offspring. The mechanism involved in skeletal myogenesis and the effect of MET supplementation during pregnancy on the maternal body remain unclear. Thus, this study aimed to investigate the potential effect of methyl donor micronutrients (MET) on skeletal muscle development and metabolism in offspring using pig models. Forty-three Duroc × Erhualian gilts were assigned to two dietary groups during gestation: control diet (CON) and CON diet supplemented with MET (folic acid, methionine, choline, vitamin B6, and vitamin B12). The results showed that maternal MET exposure during pregnancy significantly increased the concentrations of protein, triiodothyronine (T3), and thyroxine (T4) in colostrum and methyl metabolites, including S-adenosylmethionine (SAM), S-adenosyl-L-homocysteine (SAH), 5-methyl-tetrahydrofolate (5-MTHF), and betaine, in the maternal and offspring umbilical vein serum. A similar pattern was demonstrated in the body weight gain and myofiber diameters in offspring. In addition, maternal MET supplementation significantly increased the concentration of offspring serum insulin-like growth factor 1 (IGF-1), T3, and T4; upregulated the mRNA expression of IGF-1 and IGF-1 receptor (IGF-1r) and the phosphorylation level of protein kinases in offspring longissimus dorsi muscle; and upregulated the expression of myogenic genes and fast myosin heavy chain (fast MyHC) in offspring skeletal muscle. Supplementing sows with higher levels of MET during gestation may promote skeletal muscle differentiation and maturity and improve the skeletal muscle mass of the piglets.

## Introduction

Maternal nutrition during pregnancy plays a role in regulating offspring growth, development, and metabolic health through altering the epigenetic state of the genome (1–3). Previous studies showed that maternal methyl donor micronutrients (MET), such as folic acid, methionine, choline, betaine, etc., are associated with the structure, physiology, and metabolism of the offspring (4–7). MET participates in the synthesis of nucleotides, proteins, and lipids by integrating glucose, amino acid status, and vitamins and feeds into epigenetic mechanisms by methyl group donation or transfer (8). Recently, a study found that maternal MET supplementation has a significant effect on carcass traits and meat quality of pig offspring (9). The mechanism involved in skeletal myogenesis and the effect of MET supplementation during pregnancy on the maternal body remain unclear.

Skeletal muscle is one of the important peripheral tissues affected by maternal diets. It comprises ~40% of the body mass (10). The net number of skeletal muscle fibers is determined during embryogenesis, and postnatal growth is mainly determined by an increase in the fiber size. Therefore, fetal growth stages are important for skeletal muscle development and could profoundly influence postnatal skeletal muscle growth processes of the offspring (3). Myogenesis is crucial in postnatal muscle growth and regeneration (11), which needs some nutrients; the suppression factor in this process is the usability of maternal nutrients (12). Public health policies recommend periconceptional maternal supplementation of methyl donor, especially in the first trimester of pregnancy. MET is usually provided as supplements during pregnancy to promote cell division during organ growth and the development and metabolic renovation of tissues (13). Methyl donor insufficiency constitutes a risk factor for weaned pups in growth retardation, delayed ossification, and cognitive deficits (14). Previous studies have mainly focused on the impact of methyl donor on neural tube defect, metabolism, and human and mouse offspring diseases (15–17). Recently, some studies have shown that the accumulation of methyl donor in cells is beneficial in improving muscle mass and betaine has a significant effect on skeletal myogenesis, such as proliferation and differentiation of myoblast *in vitro* and *in vivo* (18). In addition, it was reported that maternal MET could regulate growth and proliferation of pig offspring, which was at least partly mediated by insulin-like growth factor 1 (IGF-1) (19). The same effect has been shown in humans (20). However, whether a high level of maternal MET is related to a healthy skeletal muscle mass in offspring remains inconclusive.

Considering the similarities in the anatomical structure and metabolism between pigs and humans, the current study used pig as a translational model for the human infant. Therefore, this study aimed to evaluate whether maternal higher MET supplementation during gestation affects the skeletal muscle developmental characteristics and muscle fiber transform of pig offspring at birth and weaning.

## Materials and Methods

### Ethical Statement

The experiment was conducted following the Chinese guidelines for animal welfare. All experimental procedures using laboratory animals were approved by the Animal Care and Use Committee of Jiangxi Agricultural University (Ethics Approval Code: JXAUA01).

### Experimental Design and Animal Material

A total of 43 gilts (Duroc × Erhualian) with initial body weight (BW) of 102.8 ± 6.3 kg and the same genetic background were artificially inseminated with purebred Duroc semen (littermate Duroc boars) and then randomly assigned to receive either a control diet (CON, *n* = 21) or a methyl donor micronutrient diet (MET, *n* = 22). Dietary treatment started at the last insemination and lasted until parturition. Ingredients and composition of pregnant gilts diets are shown in Table 1. The nutrient levels met or exceeded the recommendation of the National Research Council (NRC) (2012) (21). The difference between the two diets was that the MET supplementation was replaced by the filler (wheat middlings) in the CON diet. After producing each diet, the main dietary nutritional contents were analyzed by the near-infrared (NIR) analyzer (FOSS, Denmark). There were no differences between the two groups in crude protein, crude ash, crude fat, crude fiber, calcium, and total phosphorus. The MET-supplemented gestation diet contained 4,700 mg kg^−1^ methionine (CJ BIO, Malaysia, purity ≥99%), 16.3 mg kg^−1^ folic acid (Sigma-Aldrich, St. Louis, MO, United States, purity ≥97%), 2,230 mg kg^−1^ choline (NB GROUP, Shandong, China, purity = 60%), 0.15 mg kg^−1^ vitamin B12 (Sigma-Aldrich, purity ≥98%), and 1,180 mg kg^−1^ of vitamin B6 (Jiangxi Tianxin Pharmaceutical, Jiangxi, China, purity ≥98%). Previous reports were used as references to determine the treatment dosages of folic acid (22), vitamin B12 (9, 23), methionine, choline, and vitamin B6 (19, 24). During lactation, sows received a standard lactation diet (Table 2). Lactation lasted 24 days. The experiment began with 54 gilts, 27 gilts per treatment. Pregnancy was confirmed by ultrasonic examination 30 days post-mating; 11 gilts were eliminated due to failure of pregnancy.

**Table 1:** Ingredients and nutrient composition of diets for the pregnant sows.

**Items**	**CON**	**MET**
**Ingredients, %**		
Corn	55.00	55.00
Soybean hull	10.00	10.00
Rice bran	10.00	10.00
Expanded soybean	8.00	8.00
Soybean meal	13.00	13.00
Dicalcium phosphate	1.70	1.70
Limestone	0.20	0.20
NaCl	0.30	0.30
Lysine monohydrochloride, 98.5%	0.05	0.05
Choline chloride, 60%	0.17	0.17
Minerals^a^	0.40	0.40
Vitamins^a^	0.05	0.05
Methyl donnor^b^	0	1.11
Filler^c^	1.13	0.02
Total	100.00	100.00
**Calculated nutrient composition^b^**		
Digestible energy, Mcal kg^−1^	3.14	3.14
Crude protein, %	15.20	15.20
Total lysine, %	0.86	0.86
Standardized ideal digestible- lysine, %	0.72	0.72
Calcium, %	0.86	0.86
Total phosphorus, %	0.69	0.69
Available phosphorus, %	0.41	0.41
Folic acid, mg kg^−1^	1.30	16.30
Choline, mg kg^−1^	1025.00	2230.00
Vitamin B12, μg kg^−1^	30.00	150.00
Vitamin B6, mg kg^−1^	3.00	1180.00
Methionine, mg kg^−1^	2050.00	4700.00
**Nutrient composition, %^d^**		
Crude protein	15.26	15.22
Crude ash	5.96	5.92
Crude fat	5.63	5.57
Crude fiber	5.23	5.38
Calcium	0.94	0.98
Total phosphorus	0.64	0.64

a *Vitamin mixture supplied the following amounts of vitamins/kg of complete diet: 10,000 IU vitamin A; 1,000 IU vitamin D3; 60 IU vitamin E; 2 mg vitamin B1; 4 mg vitamin B2; 3 mg niacin; 15 mg Cu; 110 mg Fe; 100 mg Zn; 20 mg Mn; 0.2 mg I; 0.3 mg Se*.

b *Calculated according to the China Feed Database*.

c *The filler was wheat middlings (CF = 2.8%, CP = 13.6%, DE = 3.1 Mcal/kg)*.

d *The nutrient composition is measured value*.

**Table 2:** Ingredients and nutrient composition of basal diets during lactation.

**Items**	**Content**
**Ingredients %**	
Corn	59.00
Rice bran	7.00
Soybean meal	22.00
Fermented soybean meal	4.00
Soybean oil	2.00
Fish meal	2.00
Dicalcium phosphate	1.40
Limestone	1.20
NaCl	0.30
Lysine monohydrochloride, 98.5%	0.05
Choline chloride, 60%	0.17
Minerals^a^	0.20
Vitamins^a^	0.20
Filler^b^	0.48
Total	100.00
**Calculated nutrient composition^c^**	
Digestible energy, Mcal kg^−1^	3.30
Crude protein, %	18.46
Total lysine, %	1.05
Standardized ideal digestible-lysine, %	0.92
Standardized ideal digestible-methionine, %	0.27
Standardized ideal digestible-threonine, %	0.62
Standardized ideal digestible-tryptophan, %	0.20
Calcium, %	0.93
Total phosphorus, %	0.73
Available phosphorus, %	0.43
**Nutrient composition, %^d^**	
Crude protein	18.86
Crude ash	6.25
Crude fat	4.44
Crude fiber	4.05
Calcium	0.98
Total phosphorus	0.69

a *Vitamin mixture supplied the following amounts of vitamins/kg of complete diet: 7,000 IU vitamin A; 1500 IU vitamin D3; 16 IU vitamin E; 1 mg vitamin B1; 2.4 mg vitamin B2; 1 mg vitamin B6; 8 μg vitamin B12; 20 mg niacin; 0.6 mg folic acid; 16 mg Cu; 100 mg Fe; 80 mg Zn; 2 mg Mn; 0.14 mg I; 0.1 mg Se*.

b *The filler was wheat middlings (CF = 2.8%, CP = 13.6%, DE = 3.1 Mcal/kg)*.

c *Calculated according to the China Feed Database*.

d *The nutrient composition is measured value*.

During gestation, all sows were housed per group with individual feeding. Sows were fed twice per day at 08:00 and 14:00 h, and water was provided *ad libitum*. The feeding amount was based on regular breeding management of pig farm and NRC recommendations (2012). After 110 days of gestation, sows were transferred to farrowing pens. After parturition, all sows were fed on the diet three times per day (i.e., 08:00, 12:00, and 15:00 h). Freshwater was provided *ad libitum*. During the first 5 days of lactation, the amount of feed was progressively increased. Subsequently, lactating sows were fed *ad libitum* until weaning at 24 days.

### Sample Collection

After 110 days post-conception, blood samples of sows were collected via the precaval vein at 07:00 h immediately before feeding. During parturition, colostrum samples (30–40 mL) were collected from sows by hand milking after thoroughly cleaning the udder with water before newborns suckled. Besides, umbilical vein blood was collected from all sows. The colostrum and serum samples were immediately refrigerated at −20°C for further testing. The BW of each piglet was recorded after cleaning the fetal membrane. A total of 12 piglets/treatment (half male and half female) were, respectively, selected from 12 L according to the BW (as close as possible to the average BW in each treatment group). Venous blood samples were collected from piglets that were not fed on colostrum, and then the longissimus dorsi muscles were immediately collected after euthanasia, snap-frozen in liquid nitrogen, and stored at −80°C for further analyses. Longissimus dorsi samples for histological analysis were fixed in 4% paraformaldehyde in a phosphate buffer. Similarly, a total of 8 weaned piglets/treatment (half male and half female) were, respectively, selected from 8 L according to the BW (as close as possible to the average BW in each treatment group). The samples collected from the weaned piglets were similar to those of the newborn piglets.

### Serum and Colostrum Hormone Measurement

Serum from sows at day 110 and offspring umbilical cord was analyzed for S-adenosylmethionine (SAM), S-adenosyl-L-homocysteine (SAH), homocysteine (Hcy), 5-methyl-tetrahydrofolate (5-MTHF), and betaine using the enzyme-linked immunosorbent assay (ELISA) kits purchased from MLBIO (Shanghai, China). Sensitivities of the assays were 0.1 μmol/mL, 0.1 μmol/mL, 0.1 nmol/mL, 0.1 ng/mL, and 1.0 ng/mL for SAM, SAH, Hcy, 5-MTHF, and betaine, respectively. Intra- and inter-assay coefficients of variation (CV) were <10 and 15%, respectively. Serum IGF-1, triiodothyronine (T3), and thyroxine (T4) of newborn and weaning piglets were assessed using ELISA kits purchased from Nanjing Jiancheng Biotech (Nanjing, China). Sensitivities of the assays were 0.5 μg/L, 1.5 pmol/L, and 12.5 pmol/L for IGF-1, T3, and T4, respectively. The intra- and inter-assay CV was <10 and 12%, respectively.

### Colostrum Composition Analysis

All colostrum samples were analyzed for moisture, total solids (TS), solid non-fat (SNF), lactose protein, and fat content. Colostrum prolactin was assessed with ELISA kits purchased from Nanjing Jiancheng Biotech (Nanjing, China). The sensitivity of the assay was 2 ng/L, and the intra- and inter-assay CV was <10 and 12%, respectively. Colostrum immunoglobulins (Ig), T3, and T4 were determined by immunoturbidimetry and ELISA kits purchased from Sino-UK (Beijing, China). Sensitivities of the assays were 0.1 g/L, 0.1 g/L, 0.1 g/L, 0.05 ng/mL, and 1 ng/ml for immunoglobulin G (IgG), IgA, IgM, T3, and T4, respectively. The intra- and inter-assay CV was <4.5 and 9.5%, respectively, for immunoglobulins and 10 and 12%, respectively, for T3 and T4.

### Histological Analysis

Longissimus dorsi muscle samples were embedded in paraffin, sliced at a thickness of 6 μm, and stained with hematoxylin and eosin (H&E). More than ten different microscopic fields of each section were chosen to determine the cross-sectional area of muscle fibers and calculate the muscle fiber density.

### Quantitative Real-Time PCR

Total RNA was isolated from the muscle tissue using TRIzol reagent (TransGen Biotech, Beijing, China). The integrity, purity, and concentration of RNA were determined by 1% agarose gel electrophoresis and nucleic acid/protein analyzer. cDNA was synthesized with a commercial RT Master Mix kit (TransGen). qPCR amplification was performed with the BioRad PCR machine with SYBR green master mix (TransGen) following the manufacturer's guidelines. All samples were analyzed in triplicates. The optimal annealing temperature of each primer was determined. The correlation coefficients of all the standard curves were >0.99, and the amplification efficiency values were between 90 and 110% (3.6>slope>3.1). The specificity of amplification was determined by the melting curve analysis at the end of the target gene amplification. The relative mRNA expression of target genes was determined after normalization of glyceraldehyde-3-phosphate dehydrogenase (GAPDH) reference using the 2^−ΔΔCt^ method (25). The primer sequences of the target genes and GAPDH were designed by Primer 5.0 software (Premier Biosoft, Palo Alto, CA, United States) and validated by BLAST sequence alignment in NCBI (Table 3).

**Table 3:** Primer sequences of the target genes and reference genes.

**Gene**	**Primer sequence (5′-3′)**	**Product (bp)**	**GenBank ID**
IGF1	Forward: TTCAACAAGCCCACAGGGTA	102	XM_005664199.1
	Reverse: CTCCAGCCTCCTCAGATCAC		
IGF-1r	Forward: ATTACCGCAAGGGAGGGAAA	174	NM_214172.1
	Reverse: GAAGGACTTGCTCGTTGGAC		
AKT1	Forward: CTGCCCTTCTACAACCAGGA	66	HQ687753.1
	Reverse: GAAGCGGATCTCCTCCATGA		
AKT2	Forward: GTGCTTCGTGATGGAGTACG	118	HQ687754
	Reverse:C TCCAGAGCCGAGACAATCT		
IGFBP5	Forward: GTGTACCTGCCCAACTGTGA	158	NM_214099.1
	Reverse: AAGCTGTGGCACTGGAAGTC		
Myf5	Forward: AGACGCCTCAAGAAGGTCAA	95	NM_001278775.1
	Reverse: CTGAGGATCTCCACCTTGGG		
Myf6	Forward: CCCTTCAGCTACAGACCCAA	183	NM_001244672.1
	Reverse: GTCCACGATGGAAGAAAGGC		
MyOD1	Forward: GTGCAAACGCAAGACCACTA	125	NM_001002824.1
	Reverse: GATTCGGGTTGCTAGACGTG		
Myog	Forward: AATCTGCACTCCCTCACCTC	73	NM_001012406.1
	Reverse: TTTCATCTGGGAAGGCCACA		
Pax3	Forward: CAGCAGAGCAGCTTGAAGAG	152	XM_021075359.1
	Reverse: CTGCTTCCTCCATCTTGCAC		
Pax7	Forward: TGCCCTCAGTGAGTTCGATT	152	NM_001206359.1
	Reverse: ATCCAGACGGTTCCCTTTGT		
MRF4	Forward: CCCTTCAGCTACAGACCCAA	183	NM_001244672.1
	Reverse: GAGCAGCTGGAAGTAAAGGC		
MSTN	Forward: AGTGATGGCTCCTTGGAAGA	169	AY448008.2
	Reverse: TCCACAGTTGGGCCTTTACT		
TGFβ-1	Forward: GCAGGTACTCCTGGTGAACT	196	AF461808.1
	Reverse: AGGATACCAGTCGGGTAGGT		
MYH7	Forward: TTCAAGCTGGAGCTGGATGA	152	NM_213855
	Reverse: GTGAGGTCGTTGACAGAACG		
MYH2	Forward: TAGGCCCTTTGATGCCAAGA	111	NM_214136
	Reverse: GCTTCCGTCTTCACTGTCAC		
MYH1	Forward: TCAAGGACACCCAGATCCAC	166	NM_001104951
	Reverse: TCCTGTTCTGCGACTTTCCT		
MYH4	Forward: GAATCCCTGGACCAACTGGA	92	NM_001123141
	Reverse: CCTCCCTCTGCAATTTGCTC		
GAPDH	Forward: TGGAAAGGCCATCACCATCT	105	NM_001206359.1
	Reverse: ATGGTCGTGAAGACACCAGT		

*IGF1, insulin-like growth factor 1; IGF1r, insulin-like growth factor 1 receptor; AKT, protein kinases; IGFBP5, insulin growth factor-binding protein 5; Myf5, myogenic factor 5; Myf6, myogenic factor 6; MyoD1, myogenic differentiation factor 1; Myog, myogenin; Pax3, paired box gene 3; Pax7, paired box gene 7; MRF4, muscle regulatory factor 4; MSTN, myostatin; TGFβ-1, transforming growth factor-β 1; MYH, myosin heavy chain; GAPDH, glyceraldehyde-3-phosphate dehydrogenase*.

### Immunoblotting Analysis

Protein was extracted from muscle tissues with a total protein extraction kit (Beijing Solarbio Science & Technology Co., Ltd., Beijing, China) according to the manufacturer's instructions. Protein concentration was measured using the bicinchoninic acid (BCA) protein assay kit (TransGen). The total protein was then denatured with a 6× protein loading buffer (TransGen). Western blot analysis for protein kinases (t-AKT; 9272S, Cell Signaling, United States), phosphorylated protein kinases (p-AKT; 9271S, Cell Signaling, United States), myogenic differentiation factor 1 (MyoD1; 18943-1-AP, Proteintech, China), myogenin (Myog) (ab1835, Abcam, United States), slow myosin heavy chain (slow MyHC; ab11083, Abcam, United States), fast MyHC (20140-1-AP, Proteintech, China), and β-actin (66009-1-Ig, Proteintech, China) was carried out as previously described (12). Briefly, protein extracts were separated by 10% SDS-PAGE gels and transferred to a polyvinylidene difluoride (PVDF) membrane (Beijing Solarbio Science & Technology Co., Ltd., Beijing, China). The membrane was washed with TBS with 0.1% Tween 20 (TBST) and incubated with 5% non-fat dry milk in TBS with 0.1% TBST at room temperature for 60 min, incubated overnight with the selected primary antibodies at 4°C, and washed with TBST followed by incubation with either horseradish peroxidase (HRP)-conjugated goat anti-mouse or HRP-conjugated goat anti-rabbit secondary antibodies for 60 min at room temperature. Finally, membranes were visualized using the Image Lab statistical software (Bio-Rad, Laboratories, Hercules, CA, United States). The secondary antibodies were purchased from Proteintech Group (SA00001-1 and SA00001-2). The relative expressions of t-AKT, p-AKT, MyoD1, Myog, slow MyHC, and fast MyHC protein were normalized to β-actin.

### Statistical Analyses

Statistical analysis was performed using SPSS 24.0 software (SPSS Inc.). The Shapiro–Wilk test was performed to determine whether measured data sets followed a normal distribution. All data sets were normally distributed and required no transformation prior to analysis. The statistical power was adequate for detecting differences between groups. An independent-sample *t*-test was used to compare the differences between the CON and the MET. The individual sow was considered as the experimental unit for number of born and weaned piglets, and a litter was considered as the experimental unit for litter weight and daily gain weight, whereas each pig was considered as the experimental unit for the remaining data. All results are shown as means ± SD; a value of *P* < 0.05 was considered statistically significant.

## Results

### Maternal Characteristics

The reproductive performance of sows fed with the MET diet and the growth performance of offspring are shown in Table 4. There was no difference in the number of total born, born alive, and weaned piglets (*P* > 0.05). There was no significant difference in the litter weight of birth (*P* > 0.05). However, the litter weight at weaning and average daily gain were greater in piglets from the MET sows compared with those from the CON sows (*P* < 0.05).

**Table 4:** Effect of maternal methyl-donor micronutrient (MET) supplementation during gestation on growth performance of offspring (*n* = 21 per group).

**Items**	**CON**	**MET**	***P*-value**
No. of total born per litter	10.75 ± 2.14	10.92 ± 2.10	0.840
No. of born alive per litter	10.58 ± 2.07	10.69 ± 2.06	0.896
No. of weaned piglets per litter	9.00 ± 1.48	9.36 ± 1.63	0.590
Litter weight at birth, kg	13.10 ± 2.97	13.99 ± 2.05	0.391
Litter weight at weaning, kg	58.09 ± 8.27	69.40 ± 12.96	0.024
Average daily gain during 1–24 days	0.23 ± 0.03	0.26 ± 0.01	0.005

*Data are reported as means ± standard deviation. CON, control diet; MET, methyl-donor micronutrient diet*.

### Colostrum Composition of Sows

Colostrum obtained from the MET-fed sows had a higher protein content than that from the CON-fed sows (*P* < 0.05). Colostrum from the MET-fed sows tended to have a greater TS than that from the CON-fed sows (*P* = 0.069). The IgM level was increased in colostrum from the MET sows compared with that from the CON sows (*P* < 0.05). Colostrum from the MET-fed sows tended to have a greater IgG level than that from the CON-fed sows (*P* = 0.060). T3 and T4 concentrations were significantly higher in the MET group colostrum compared with the CON group colostrum (*P* < 0.05). However, the ratio of T3 to T4 did not differ in the colostrum of the CON and the MET sows (*P* > 0.05; Table 5). Moreover, serum prolactin was higher in the supplemented sows than in the control sows (*P* < 0.05). However, there was no significant difference in colostrum prolactin between these two groups (*P* > 0.05; Table 6).

**Table 5:** Effect of maternal MET supplementation during gestation on nutrition composition, immunoglobulin, total triiodothyronine (T3), and thyroxine (T4) concentrations in colostrum (*n* = 10 per group).

**Items**	**CON**	**MET**	***P*-value**
**Nutrition composition, %**			
Moisture	73.96 ± 2.77	72.45 ± 4.23	0.377
TS	13.89 ± 2.21	16.96 ± 3.78	0.069
SNF	8.71 ± 0.49	8.74 ± 0.55	0.922
Fat	3.27 ± 0.31	3.36 ± 0.38	0.603
Lactose	3.02 ± 0.33	2.91 ± 0.37	0.493
Protein	5.49 ± 0.80	6.88 ± 1.36	0.018
**Colostrum immunoglobulins, ng/L**			
IgM	3.18 ± 0.25	3.88 ± 0.59	0.023
IgG	60.75 ± 6.38	67.34 ± 3.68	0.060
IgA	11.11 ± 0.79	11.82 ± 0.82	0.156
**Colostrum hormones**			
T3, ng/mL	0.12 ± 0.01	0.15 ± 0.02	0.005
T4, ng/mL	4.49 ± 0.94	5.63 ± 0.57	0.007
T3/T4	0.03 ± 0.01	0.03 ± 0.00	0.177

*Data are reported as means ± standard deviation. TS, total solids; SNF, solid-non-fat; Ig, immunoglobulin CON, control diet; MET, methyl-donor micronutrient diet*.

**Table 6:** Effect of maternal MET supplementation during gestation on prolactin concentration in sows' serum and colostrum (*n* = 10 per group).

**Items**	**CON**	**MET**	***P*-value**
Serum prolactin, ng/L	220.85 ± 37.20	283.32 ± 52.70	0.034
Colostrum prolactin, ng/L	102.46 ± 35.01	137.11 ± 9.60	0.099

*Data are reported as means ± standard deviation. CON, control diet; MET, methyl-donor micronutrient diet*.

### Serum Methyl Metabolite Profile

There was a significantly higher concentration of SAM in the sows and offspring umbilical vein of the MET group compared with the CON group (*P* < 0.05; Table 7). Similarly, serum concentrations of SAH, 5-MTHF, and betaine in the sows and offspring umbilical vein were higher in the treated group than in the control group (*P* < 0.05). However, the Hcy concentration in the sows and offspring umbilical vein serum was significantly decreased in the MET group (*P* < 0.05).

**Table 7:** Effect of maternal MET supplementation during gestation on the concentration of S-adenosylmethionine (SAM), S-adenosyl-L-homocysteine (SAH), homocysteine (Hcy), 5-methyl-tetrahydrofolate (5-MTHF), and betaine in sows and offspring umbilical vein serum (*n* = 10 per group).

**Items**	**CON**	**MET**	***P*-value**
**Sows**			
SAM, μmol/mL	41.98 ± 10.80	73.63 ± 15.86	0.004
SAH, μmol/mL	23.76 ± 5.83	37.17 ± 7.06	0.006
Hcy, nmol/mL	6.66 ± 1.57	4.42 ± 0.66	0.005
5-MTHF, ng/mL	19.45 ± 8.51	30.75 ± 6.93	0.030
Betaine, ng/L	1.01 ± 0.29	2.13 ± 0.45	0.001
**Offspring umbilical vein**			
SAM, μmol/mL	20.16 ± 1.06	22.67 ± 1.89	0.032
SAH, μmol/mL	11.19 ± 0.69	12.24 ± 0.36	0.001
Hcy, nmol/mL	17.15 ± 1.53	15.01 ± 1.26	0.033
5-MTHF, ng/mL	3.70 ± 0.22	3.99 ± 0.35	0.037
Betaine, ng/L	0.37 ± 0.03	0.42 ± 0.05	0.003

*Data are reported as means ± standard deviation. CON, control diet; MET, methyl-donor micronutrient diet*.

### Serum Hormone Profile

Maternal MET supplementation during gestation increased serum IGF-1 concentration in the offspring at birth (*P* < 0.05; Table 8). However, there was no significant difference in serum IGF-1 concentration in the weaning pigs (*P* > 0.05). There was a significantly higher concentration of serum T3 and T4 in the weaning offspring of the MET group compared with the CON group (*P* < 0.05). The serum T3 concentration in piglets at birth and weaning was increased in the MET group (*P* = 0.066, *P* < 0.05, respectively). Furthermore, the maternal MET diet increased the ratio of T3 to T4 in the offspring serum at birth and weaning (*P* < 0.05).

**Table 8:** Effects of maternal MET supplementation during gestation on serum concentration of insulin-like growth factor 1 (IGF-1), triiodothyronine (T3), and thyroxine (T4) in offspring (*n* = 12 at birth; *n* = 8 at weaning).

**Items**	**CON**	**MET**	***P*-value**
**Birth**			
IGF-1, μg/mL	23.76 ± 2.02	25.37 ± 1.37	0.048
T3, ng/mL	0.22 ± 0.02	0.36 ± 0.06	0.066
T4, ng/mL	0.99 ± 0.27	1.50 ± 0.20	0.001
T3/T4	0.20 ± 0.04	0.16 ± 0.02	0.035
**Weaning**			
IGF-1, μg/mL	26.17 ± 1.80	26.75 ± 2.06	0.569
T3, ng/mL	0.27 ± 0.03	0.38 ± 0.06	0.010
T4, ng/mL	1.15 ± 0.27	2.10 ± 0.24	<0.001
T3/T4	0.24 ± 0.05	0.16 ± 0.02	0.033

*Data are reported as means ± standard deviation. CON, control diet; MET, methyl-donor micronutrient diet*.

### Muscle Histological Analysis

**Figure 1:**
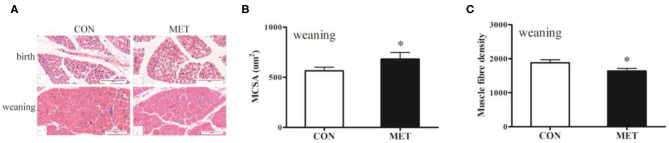
Effects of maternal MET supplementation during gestation on histological properties in offspring longissimus dorsi muscle. Data are reported as means ± standard deviation, *n* = 12 at birth; *n* = 8 at weaning. **(A)** Representative hematoxylin and eosin (H&E) staining of newborn and weaning piglets. **(B)** Number of muscle fibers per mm^2^ of weaning offspring. **(C)** Muscle fiber cross-sectional area of weaning offspring. CON, control diet; MET, methyl-donor micronutrients diet. *P < 0.05 (significant differences between MET vs. CON).

To determine whether MET exposure altered offspring skeletal muscle fiber size, we evaluated the cross-sectional fiber area of the longissimus dorsi muscle. H&E staining showed that the diameter of the muscle fiber of the weaning pigs in the MET group was thicker than that of the CON group (Figure 1). The number of muscle fibers was decreased, and the muscle fiber cross-sectional area was increased in the weaning pigs with maternal MET dietary supplementation (*P* < 0.05). However, there was no variation in the offspring at birth.

### Expression of Muscle IGF Signaling Gene

**Figure 2:**
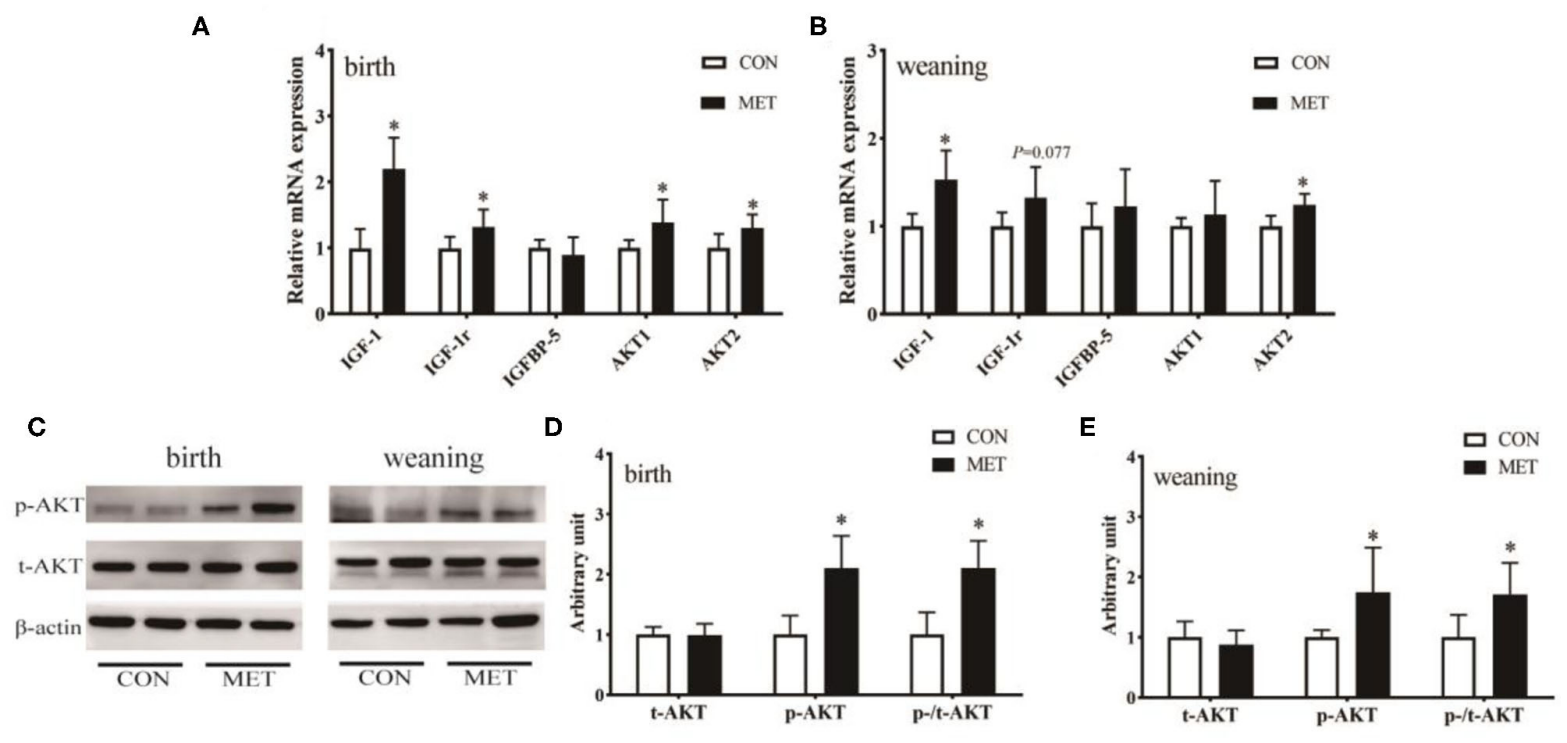
Effects of maternal MET supplementation during gestation on IGF system gene mRNA expression and t-AKT and p-AKT protein expression in offspring longissimus dorsi muscle. Data are reported as means ± standard deviation, *n* = 12 at birth; *n* = 8 at weaning. **(A)** IGF system gene mRNA expression in newborn piglets. **(B)** IGF system gene mRNA expression in weaning piglets. **(C)** Representative images of immunoblotting. **(D)** Immunoblotting analysis of t-AKT and p-AKT protein expression in newborn piglets. **(E)** Immunoblotting analysis of t-AKT and p-AKT protein expression in weaning piglets. t-AKT, protein kinases; p-AKT, phosphorylated protein kinases; IGF1, insulin-like growth factor 1; IGF1r, insulin-like growth factor 1 receptor; IGFBP5, insulin growth factor-binding protein 5; GAPDH, glyceraldehyde-3-phosphate dehydrogenase; t, total content, p, phosphorylated; CON, control diet; MET, methyl-donor micronutrient diet. *P < 0.05 (significant differences between MET vs. CON).

To further explore why maternal MET supplementation increased serum IGF-1 concentration in offspring at birth, we measured muscle IGF system genes mRNA expression and t-AKT and p-AKT protein expression in offspring at birth and weaning (Figure 2). Maternal dietary treatment had a significant effect on the mRNA abundance of the IGF signaling genes, including IGF-1 (*P* < 0.05), IGF-1r (*P* < 0.05), AKT1 (*P* < 0.05), and AKT2 (*P* < 0.05). A similar pattern was also demonstrated for gene expression of the longissimus dorsi muscle IGF-1 (*P* < 0.05) and AKT2 (*P* < 0.05) in the weaning pigs. Although the expression of total AKT protein level was not different, its phosphorylation was higher (*P* < 0.05) in MET supplemented offspring when compared to the CON group; the MET offspring had a significantly higher p-/t-AKT ratio (*P* < 0.05).

### Expression of Muscle Growth-Related Genes

**Figure 3:**
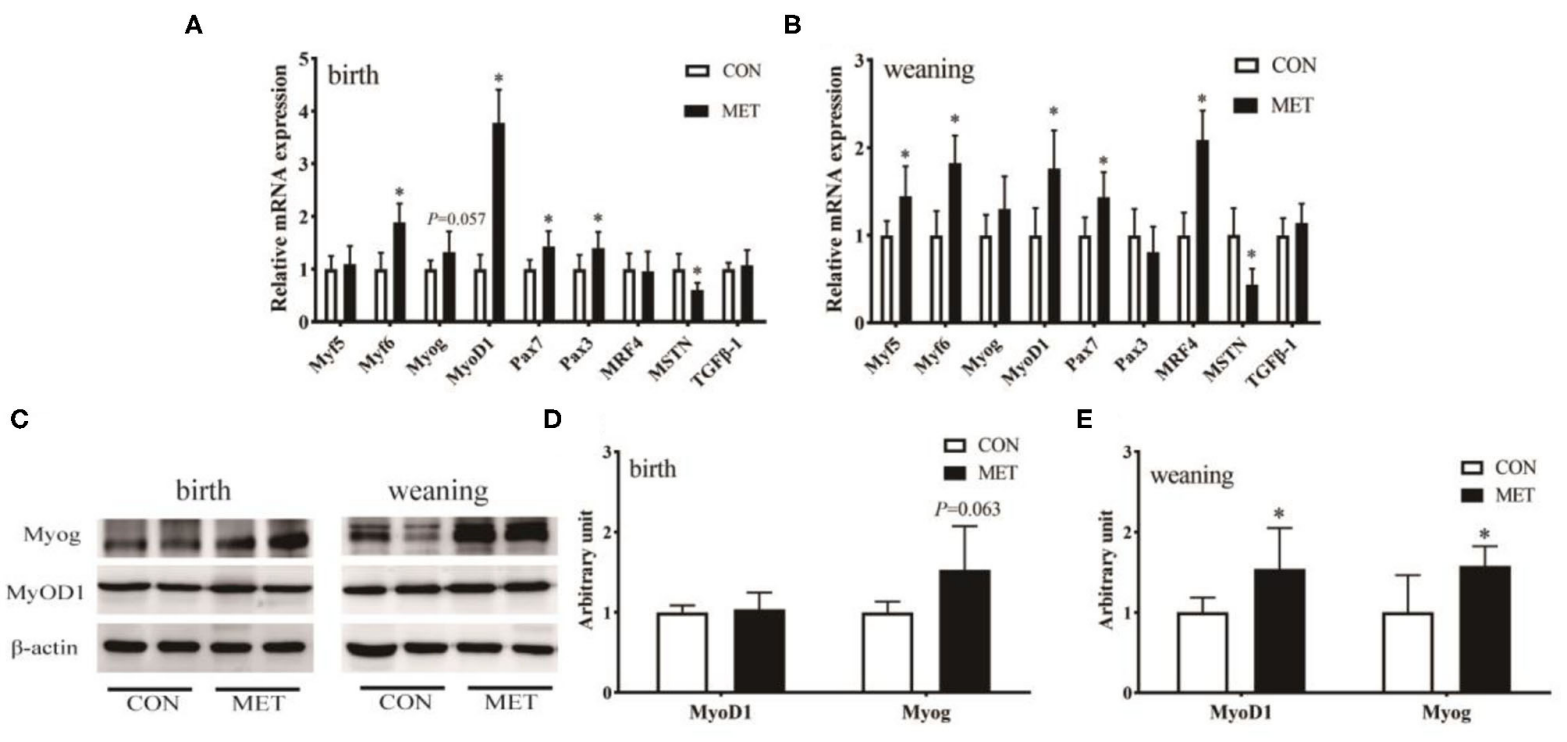
Effects of maternal MET supplementation during gestation on longissimus dorsi muscle growth-related genes mRNA expression and MyoD1 and Myog protein expression in offspring. Data are reported as means ± standard deviation, *n* = 12 at birth; *n* = 8 at weaning. **(A)** Growth-related gene mRNA expression in newborn piglets. **(B)** Growth-related gene mRNA expression in weaning piglets. **(C)** Representative images of immunoblotting. **(D)** Immunoblotting analysis of MyoD1 and Myog protein expression in newborn piglets. **(E)** Immunoblotting analysis of MyoD1 and Myog protein expression in weaning piglets. MyoD1, myogenic differentiation factor 1; Myog, myogenin; Myf5, myogenic factor 5; Myf6, myogenic factor 6; Pax3, paired box gene 3; Pax7, paired box gene 7; MRF4, muscle regulatory factor 4; MSTN, myostatin; TGFβ-1, transforming growth factor-β 1; GAPDH, glyceraldehyde-3-phosphate dehydrogenase; CON, control diet; MET, methyl-donor micronutrient diet. *P < 0.05 (significant differences between MET vs. CON).

The mRNA abundance of the longissimus dorsi muscle growth-related genes is shown in Figures 3A,B. Dietary MET supplementation increased the mRNA expression of myogenic factor 6 (Myf6; *P* < 0.05), MyoD1 (*P* < 0.05), and paired box gene 7 (Pax7; *P* < 0.05) and reduced the mRNA expression of myostatin (MSTN; *P* < 0.05). The mRNA expression of Pax3 (*P* < 0.05) and Myog (*P* = 0.057) was increased in the longissimus dorsi muscle of MET compared to the CON newborn piglets. Maternal MET supplementation significantly upregulated the mRNA expression of Myf5 (*P* < 0.05) and muscle regulatory factor 4 (MRF4; *P* < 0.05) in the weaning offspring. Western blotting further indicated that the protein expression of MyoD1 and Myog (*P* < 0.05) was significantly increased in the weaning offspring with maternal exposure to MET (Figures 3C–E). In addition, Myog protein expression of the newborn offspring tended to be increased in the MET group (*P* = 0.063).

### Expression of Muscle Fiber Type-Related Genes

**Figure 4:**
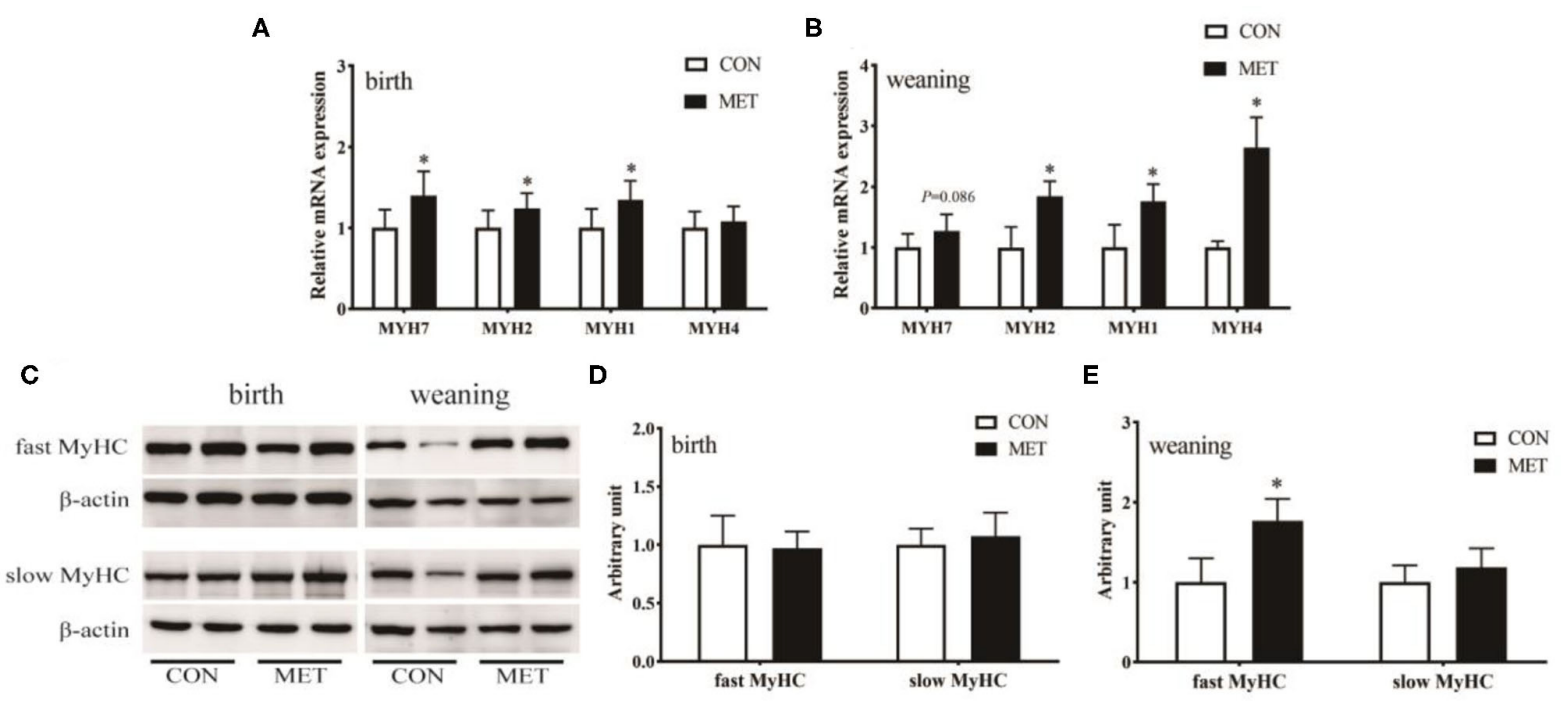
Effects of maternal MET supplementation during gestation on longissimus dorsi muscle fiber type-related genes mRNA expression and fast MyHC and slow MyHC protein expression in offspring. Data are reported as means ± standard deviation, *n* = 12 at birth; *n* = 8 at weaning. **(A)** Muscle fiber type-related gene *n* = 12 at birth; *n* = 8 at weaning. **(A)** Muscle fiber expression in weaning piglets. **(C)** Representative images of immunoblotting. **(D)** Immunoblotting analysis of fast MyHC and slow MyHC protein expression in newborn piglets. **(E)** Immunoblotting analysis of fast MyHC and slow MyHC protein expression in weaning piglets. MYH/MyHC, myosin heavy chain; GAPDH, glyceraldehyde-3-phosphate dehydrogenase; CON, control diet; MET, methyl-donor micronutrients diet. *P < 0.05 (significant differences between MET vs. CON).

The mRNA abundance of MYH7 (*P* < 0.05), MYH2 (*P* < 0.05), and MYH1 (*P* < 0.05) was increased in the longissimus dorsi muscle in the MET compared to the CON newborn piglets (Figure 4A). Prenatal MET exposure increased the mRNA expression of MYH2 (*P* < 0.05), MYH1 (*P* < 0.05), and MYH4 (*P* < 0.05) in the weaning offspring (Figure 4B). Consistent with their mRNA expression, there was no significant difference in the protein expression of the fast MyHC isoform in the newborn piglets (*P* > 0.05). However, the protein expression of the fast MyHC isoform in weaning piglets was upregulated in the treated group than in the control group (*P* < 0.05; Figures 4C–E).

## Discussion

In our study, maternal MET exposure during pregnancy increased protein, T3, and T4 concentrations in colostrum and methyl metabolites, including SAM, SAH, 5-MTHF, and betaine, in maternal and offspring umbilical vein serum. Similar findings were recorded in offspring BW gain and myofiber diameters. Meanwhile, maternal MET supplementation significantly increased the concentration of serum IGF-1, T3, and T4 in the offspring, decreased the concentration of maternal and offspring umbilical vein serum Hcy, and upregulated the mRNA expression of IGF-1 and IGF-1r and the phosphorylation level of AKT in the longissimus dorsi muscle of the offspring. Furthermore, maternal exposure to MET during pregnancy promoted skeletal muscle differentiation and maturity in pig offspring. We found no differences in the reproduction performance of the sows, which was similar to findings from a study by Zhao et al. (9). Some studies also found either a negative or no relationship between MET and fetal growth (26, 27). In betaine-supplemented dams, the first filial generation litter weights were smaller at birth and weaning, whereas the second filial generation had exactly the reverse effect in weaning (6). To date, the effect of maternal MET exposure during gestation on the growth performance of pig offspring has been controversial. However, maternal nutrition during pregnancy has been found to affect offspring development and has a profound effect throughout the life of the offspring via epigenetic modifications (28, 29). Thus, we speculate that the main reason for the different results is the different types and doses of MET.

Colostrum plays a major role in the survival of piglets during farrowing by not only providing heat and metabolic energy but also preventing infections through passive immunity. However, there are many factors affecting colostrum production, including genotype, parity, age, nursing behavior, and litter characteristics (30). To minimize the potential impact of sow condition on lactation performance, we used primiparous sow with similar genetic background and BW. Previously, it was reported that low-colostrum-producing sows have greater concentrations of progesterone and colostrum production in primiparous sows was positively associated with prolactin concentration (31). In our study, serum obtained from MET-fed sows had higher prolactin content than that obtained from CON-fed sows, which means sows could produce more milk to meet the needs of piglets. Meanwhile, colostrum obtained from MET-fed sows had higher protein content than that obtained from CON-fed sows. Because colostrum components are synthesized as early as a few weeks before farrowing, milk composition in colostrum is largely dependent on maternal body conditions (32). Previous studies have reported that methionine is an essential amino acid that may play a major role in swine protein structure and metabolism (33) and maintain the health of the sows (34). Methionine is a potential regulator of milk protein synthesis; it may easily restrict mammary protein synthesis (35). Furthermore, MET participates in the synthesis of nucleotides, proteins, and lipids by integrating glucose, amino acid status, and vitamins and feeds into epigenetic mechanisms by methyl group donation or transfer (8). These are also the main reasons for the increase in colostrum protein. Colostrum contains both nutrients and bioactive molecules. The concentration of the bioactive molecule in colostrum is closely related to the metabolic status of the sow during the perinatal and postnatal periods. We found higher T3, T4, and IgM in colostrum from MET-fed sows. Maternal MET exposure during pregnancy may promote mammary gland development of gilt and further increase the amount and quality of lactation, which lead to the offspring getting more adequate nutrition, promote skeletal muscle differentiation and maturity, and, ultimately, improve the growth performance of the offspring.

Previously, it was reported that an imbalance of methionine content in maternal diet will reduce the postnatal growth in rats (36). If the supply of methionine exceeds the maximum amount the animal can withstand, it may cause toxicities, such as hyperhomocysteinemia (a thrombogenic non-proteinogenic amino acid) and endothelial dysfunction. Hcy is a demethylated derivative of methionine that is produced during the one-carbon metabolism cycle. SAM is the most important metabolic substrate in methylation and participates in most methylation in animals (37). We found significantly higher serum SAM and SAH concentrations in sows and offspring umbilical vein of the MET group compared with the CON group. Folic acid supplementation was effective in reducing the total homocysteine levels (38). Reduced levels of serum Hcy in MET sows and the newborn pigs may be related to the increase in serum 5-MTHF concentration, which is the substrate of Hcy remethylation to methionine (39).

Dietary supplementation of methyl donors has been shown to promote Hcy metabolism. Moreover, changes in serum Hcy have been negatively correlated with IGF-I (40). Growth hormone (GH) stimulates protein synthesis, and IGFs are essential for the rapid proliferation and differentiation of most tissues in animals (41). Furthermore, skeletal muscle is a major insulin target. The thicker muscle fiber diameter of the weaning offspring demonstrates that maternal MET exposure may improve the growth and formation of offspring fibers. We speculated that the offspring growth might be associated with muscle IGF signaling because maternal MET supplementation significantly increased the serum IGF-1 level of the offspring at birth. Additionally, we found that maternal exposure to MET increased offspring serum T3 and T4 concentrations, which are major factors in piglet muscle development (42). Moreover, we found a significantly upregulated mRNA expression of IGF-1 in offspring longissimus dorsi muscle in the MET group. Protein expression indicated that MET offspring had a significantly higher p-/t-AKT ratio. AKT activation negatively regulates numerous pro-apoptotic factors and positively regulates the transcription factor, MyoD, in myoblast cells, inducing terminal differentiation into myocytes and then fusion to regenerate myofibers (43, 44).

Myogenesis is a multistep process, including myoblast proliferation and differentiation, from precursor determination to patterning, differentiation, fusion, and maturation of muscle fiber types (45). It is crucial for muscle growth and regeneration after delivery. Pax3 and Pax7 are important regulators of myogenic progenitor cells, which can directly act upstream of myogenic regulatory factors (MRFs) to further promote muscle differentiation and growth (46, 47). MRFs include MyoD1, Myf5, Myog, and MRf4 (48). MyoD1 and Myf5 are involved in muscle determination, while Myog plays a predominant role in muscle cell differentiation to form muscle fibers. MRF4 is essential in the maintenance of postnatal muscles (49). MSTN is a negative regulator of skeletal muscle growth (50). After birth, muscle growth and development are mainly based on the increase of the muscle fiber area (51), Myogenesis promotes muscle fiber hypertrophy and growth by maintaining the proliferation and differentiation of the muscle satellite cells (3). The present results showed that the diameter of muscle fiber in weaning pigs in the MET group was thicker than that in the CON group. Furthermore, the mRNA expression of Myf5, MyoD1, MRF4, Myog, and Pax7 was increased and the protein expression of Myog was upregulated in weaning pigs in the MET group, suggesting that MET supplementation during gestation improved myogenesis and promoted muscle fiber hypertrophy and growth. We also noted that maternal MET supplementation during gestation increased MRF4 mRNA in weaning offspring. This was similar to findings from a previous study, which showed that MRF4 expression increased during hypertrophy induced by a high level of methionine administration in mice (52). However, although we did not measure the levels of MRF4 protein, it is tempting to infer that the increased level of MRF4 mRNA was probably associated with postpartum skeletal muscle hypertrophy and growth upon MET administration in sows.

Generally, skeletal muscle fiber types are characterized in part by the expression of multiple MYH genes (53). Muscle fiber types can be classified into slow MyHC and fast MyHC. We found that gestational MET increased MYH2, MYH1, and MYH4 mRNA expression and upregulated fast MyHC protein expression in weaning offspring, indicating that gestational MET caused early muscle differentiation leading to a higher degree of muscular maturity during weaning. Consistent with Senesi's et al. (54) findings, betaine supplementation enhanced skeletal muscle differentiation via IGF activation. The increased fast-twitch myofiber isoform in MET weaning piglets was also linked to serum T3 and T4 in our study. The thyroid hormone is a major determinant of myofiber composition, which can promote the uptake and utilization of glucose by muscle tissue and regulate the conversion of embryonic or prenatal MyHC isoforms to adult fast MyHC isoforms (42). Muscle differentiation is a process from slow-twitch myofibers to fast-twitch myofibers. MET regulates the expression of myogenesis-related genes through DNA methylation, thereby regulating the proliferation and differentiation of myoblast (55). It can significantly increase the concentrations of 5-mC and 6-mA than affect DNA methylation levels in C2C12 myoblasts (18, 56). However, we failed to measure the degree of methylation of the gene promoter. Nonetheless, according to the number of methyl metabolites and gene expression, we speculate that maternal MET exposure during pregnancy may promote skeletal muscle differentiation and maturity in offspring through the upregulation of the muscle gene expression of IGFs.

## Conclusion

In summary, maternal MET exposure during pregnancy promoted the differentiation and maturity of skeletal muscle in pig offspring, which was associated with improved colostrum quality (protein, Ig, T3, and T4); a higher concentration of IGF-1, T3, SAM, SAH, and 5-MTHF; and lower Hcy level in offspring serum and upregulated expression of myogenic genes and fast MyHC in offspring skeletal muscle. These results suggest that supplementing sows with higher MET during gestation can promote skeletal muscle differentiation and maturity and improve the skeletal muscle mass of piglets.

## Data Availability Statement

The original contributions presented in the study are included in the article/supplementary material, further inquiries can be directed to the corresponding author/s.

## Ethics Statement

The animal study was reviewed and approved by the experiment was conducted following the Chinese guidelines for animal welfare. All experimental procedures using laboratory animals were approved by the Animal Care and Use Committee of Jiangxi Agricultural University (Ethics Approval Code: JXAUA01).

## Author Contributions

JY and TZ contributed to experimental concepts and design. QH, LJ, and JH performed the animal feeding experiment and sample analysis. ZW, FX, and YX assisted with immunoblotting analysis and the detection of the serum level of hormones. QH analyzed the data and wrote the manuscript. JY, TZ, and JC finalized the manuscript. All authors read and approved the final manuscript.

## Conflict of Interest

The authors declare that the research was conducted in the absence of any commercial or financial relationships that could be construed as a potential conflict of interest.
